# Integration of silver nanostructures in wireless sensor networks for enhanced biochemical sensing

**DOI:** 10.1186/s11671-024-04159-6

**Published:** 2025-01-13

**Authors:** M. Sahaya Sheela, S. Kumarganesh, Binay Kumar Pandey, Mesfin Esayas Lelisho

**Affiliations:** 1 Department of ECE, Vel Tech Rangarajan Dr. Sagunthala R&D Institute of Science and Technology, Chennai, Tamil Nadu India; 2 Department of ECE, Knowledge Institute of Technology, Salem, Tamil Nadu India; 3 Department of Information Technology, College of Technology, Govind Ballabh Pant University of Agriculture and Technology Pantnagar, Udham Singh Nagar, India; 4 Mizan-Tepi University, Tepi, Ethiopia

**Keywords:** Nanostructures, Sensor, Sodium borohydride, Capping agents, Biochemical sensing, Polyvinyl alcohol, Polyethylene glycol

## Abstract

Integrating noble metal nanostructures, specifically silver nanoparticles, into sensor designs has proven to enhance sensor performance across key metrics, including response time, stability, and sensitivity. However, a critical gap remains in understanding the unique contributions of various synthesis parameters on these enhancements. This study addresses this gap by examining how factors such as temperature, growth time, and choice of capping agents influence nanostructure shape and size, optimizing sensor performance for diverse conditions. Using silver nitrate and sodium borohydride, silver seed particles were created, followed by controlled growth in a solution containing additional silver ions. The size and morphology of the resulting nanostructures were regulated to achieve optimal properties for biochemical sensing in wireless sensor networks. Results demonstrated that embedding these nanostructures in Polyvinyl Alcohol (PVA) matrices led to superior stability, maintaining 93% effectiveness over 30 days compared to 70% in Polyethylene Glycol (PEG). Performance metrics revealed significant improvements: reduced response times (1.2 ms vs. 1.5 ms at zero analyte concentration) and faster responses at higher analyte levels (0.2 ms). These outcomes confirm that higher synthesis temperatures and precise shape control contribute to larger, more stable nanostructures.The enhanced stability and responsiveness underscore the potential of noble metal nanostructures for scalable and durable sensor applications, offering a significant advancement over current methods.

## Introduction

Biochemical sensing is essential across fields like healthcare, environmental monitoring, food safety, and biotechnology, as it detects biological or chemical substances to provide crucial data. In healthcare, these sensors support diagnostics, disease monitoring, and treatment, with glucose sensors for diabetes management and biosensors for early detection of conditions like cancer, enhancing patient outcomes [1]. Wearable sensors that monitor vital signs, such as heart rate and oxygen levels, further support real-time health monitoring and preventive care. Environmental monitoring leverages biochemical sensing to detect pollutants, toxins, and pathogens in air, water, and soil, helping protect ecosystems and public health [2]. For example, water biosensors can identify harmful bacteria, ensuring safe drinking water, and air quality sensors detect hazardous gases, helping prevent respiratory diseases. In agriculture, biochemical sensors monitor soil health and crop conditions, promoting efficient resource use and yield improvement. Food safety benefits from biochemical sensing by identifying contaminants, such as pathogens, toxins, and allergens, preventing foodborne illnesses and maintaining quality standards [3]. Sensors detecting E. coli or Salmonella in food ensure public health protection and reduce waste by monitoring freshness [4]. Biotechnology applications rely on biochemical sensing for drug development, genetic analysis, and cellular research. Sensors like enzyme-linked immunosorbent assays (ELISAs) enable precise protein and antibody quantification, which is vital in immunology and disease research [5].

Integrating biochemical sensing with wireless sensor networks (WSNs) enhances monitoring capabilities and efficiency. WSNs consist of spatially distributed wireless sensors, allowing real-time, continuous monitoring over large areas [6]. This is valuable in environmental monitoring, where early contamination detection enables rapid response [7]. In healthcare, combining WSNs with biochemical sensors allows wearable devices to continuously monitor vital signs and biochemical markers, like glucose, with real-time data transmission for timely intervention and personalized care, eliminating invasive procedures [8]. Food safety applications benefit from WSNs by tracking conditions such as temperature, humidity, and contaminants throughout production and distribution, preventing spoilage and reducing foodborne illness risks [3]. Additionally, in agriculture, WSN-integrated biochemical sensors optimize resource use and productivity by monitoring soil health, nutrients, and crop status, leading to efficient practices and improved yields [3].

## Literature review

Electrochemical sensors, such as potentiometric and amperometric sensors, have been widely used in biochemical sensing due to their high sensitivity and specificity. Potentiometric sensors measure changes in voltage due to biochemical reactions and are commonly used for pH sensing and ion-selective electrodes [9]. Amperometric sensors, on the other hand, measure the current produced by oxidation or reduction reactions, making them ideal for applications like glucose monitoring and detecting environmental pollutants. These sensors offer real-time monitoring capabilities and are relatively easy to miniaturize for integration with WSNs. Despite their advantages, electrochemical sensors face challenges such as susceptibility to interference from other chemical species, limited stability over time, and the need for frequent calibration [10]. Additionally, their performance can be affected by changes in temperature and pH, which can lead to inaccurate readings. The requirement for a continuous power supply for data transmission in WSNs also poses a challenge in remote or resource-limited settings [11].

Optical sensors, including absorbance spectroscopy and fluorescence spectroscopy, detect biochemical substances through their interaction with light. Absorbance spectroscopy measures the amount of light absorbed by a sample at specific wavelengths, which is useful for detecting various biomolecules [12]. Fluorescence spectroscopy uses fluorescent markers that emit light upon excitation, providing high sensitivity for detecting low concentrations of analytes. Surface plasmon resonance (SPR) is another optical method that measures changes in the refractive index near the sensor surface, useful for studying molecular interactions and detecting biomolecules [13]. Optical sensors, while highly sensitive, often require complex and bulky instrumentation, making miniaturization for WSN integration challenging. They can also be affected by environmental factors such as ambient light, temperature fluctuations, and the presence of other fluorescent substances, which can interfere with accurate measurements. Additionally, the need for external light sources and sophisticated detection systems can increase power consumption and complexity in WSN applications [14].

Piezoelectric sensors, like quartz crystal microbalance (QCM), operate based on the principle that certain materials generate an electrical charge in response to mechanical stress [15]. These sensors can detect mass changes on a sensor surface by measuring the frequency shift of a quartz crystal, making them useful for studying protein–ligand interactions and environmental monitoring. QCM sensors are valued for their high sensitivity and ability to provide real-time monitoring of molecular interactions [16]. The primary limitation of piezoelectric sensors is their sensitivity to environmental factors such as temperature and humidity, which can cause drift and affect accuracy. Additionally, these sensors often require sophisticated signal processing and calibration to maintain performance. Integrating piezoelectric sensors with WSNs can be challenging due to their relatively high power consumption and the need for stable environmental conditions to ensure reliable data transmission and sensor operation [17]. Table 1 provides a comparison of several current sensing methods for the biochemical application.

**Table 1:** Comparison of different sensing techniques for biochemical applications

Sensing technique	Advantages	Disadvantages
Potentiometric sensors	High sensitivity, useful for pH sensing, ion detection	Susceptible to interference, limited stability, requires frequent calibration
Amperometric sensors	Ideal for oxidation/reduction-based detection, real-time monitoring	Affected by changes in temperature and pH, requires continuous power supply
Optical sensors	High sensitivity, versatile in biomolecule detection	Complex instrumentation, bulky setups, interference from ambient light
Piezoelectric sensors	Real-time monitoring, sensitive to mass changes	Environmental sensitivity (temperature, humidity), drift issues

## Proposed work

### Seed formation and growth mechanism

The implementation of silver nanostructure synthesis begins with the creation of small silver seed particles, which serve as nucleation sites. The initial step typically involves the reduction of a silver nitrate solution using a strong reducing agent, such as sodium borohydride. This reduction process results in the formation of tiny silver seed particles. These initial seed particles are crucial as they act as the foundational nuclei upon which further silver deposition can occur during the subsequent growth phase. Once the seed particles have been successfully formed, they are introduced into a growth solution that contains additional silver ions and a milder reducing agent, such as ascorbic acid. The conditions of the growth solution are meticulously controlled to ensure that the additional silver ions deposit onto the existing seed particles rather than nucleating new particles. This precise control over the deposition process is essential for the growth of the seed particles into larger and well-defined nanostructures.

**Fig. 1:**
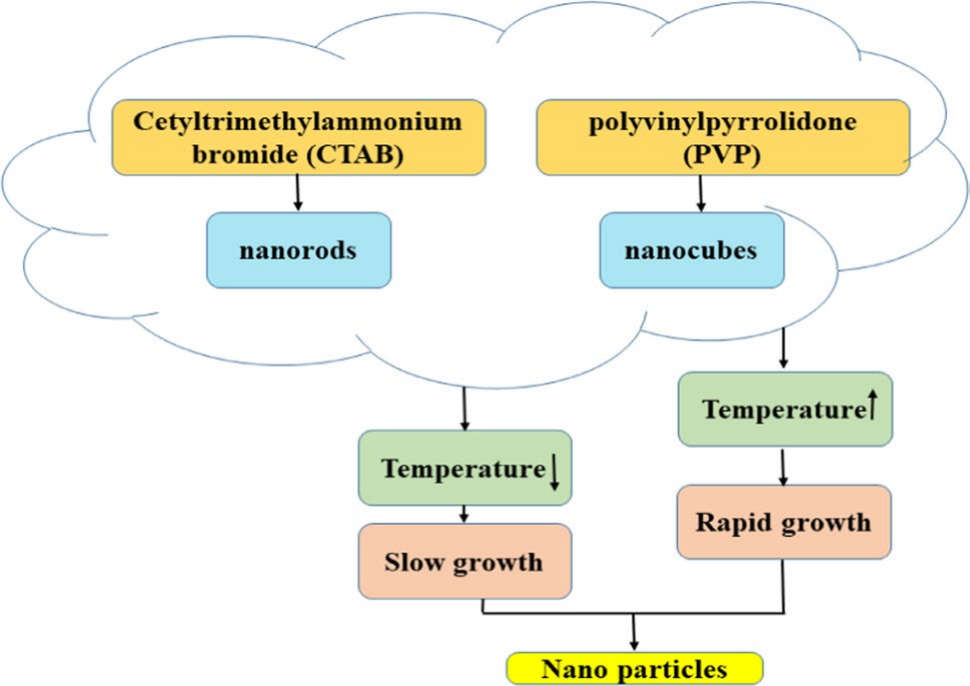
Factors influencing the growth and shape of silver nanostructures

The growth of the seed particles into larger nanostructures can be finely tuned by adjusting several parameters as shown in Fig. 1. These parameters include the concentration of silver ions in the growth solution, the type and concentration of the reducing agent, and the presence of specific capping agents. The selection of specific capping agents and polymer matrices is critical for optimizing sensor performance characteristics. Capping agents, such as cetyltrimethylammonium bromide (CTAB), influence the morphology of nanostructures, impacting surface area and reactivity [21]. For example, CTAB promotes the formation of nanorods, which can enhance surface plasmon resonance effects, improving sensitivity for optical sensors. On the other hand, polymer matrices provide a supportive environment for nanostructures, influencing mechanical stability, conductivity, and interaction with target analytes. Polymers like polyvinyl alcohol (PVA) or polystyrene can be selected for their favorable properties, such as flexibility and ease of processing. The combined choice of capping agents and polymers is aimed at achieving optimal dispersion, stability, and enhanced sensitivity, thereby ensuring efficient sensor performance. However, this rapid growth can sometimes lead to less uniform shapes. Conversely, lower temperatures can slow down the growth process, allowing for more controlled and uniform particle formation. The duration of the growth reaction also influences the final nanostructures. Longer reaction times provide more opportunities for silver ions to deposit onto the seed particles, which can lead to more extensive growth and potentially enable shape transformations.

The experimental plan will commence with testing various concentrations of silver nitrate and sodium borohydride to optimize the initial seed formation phase. Growth phases will use ascorbic acid under varied temperature and time conditions (20–100 min) to observe impacts on particle size and uniformity. Experiments will include multiple trials with CTAB and PVA as capping agents, alongside comparisons with alternative polymers, to assess differences in sensor performance. Reproducibility will be confirmed by conducting triplicate experiments and measuring consistency in particle morphology and optical properties.

**Fig. 2:**
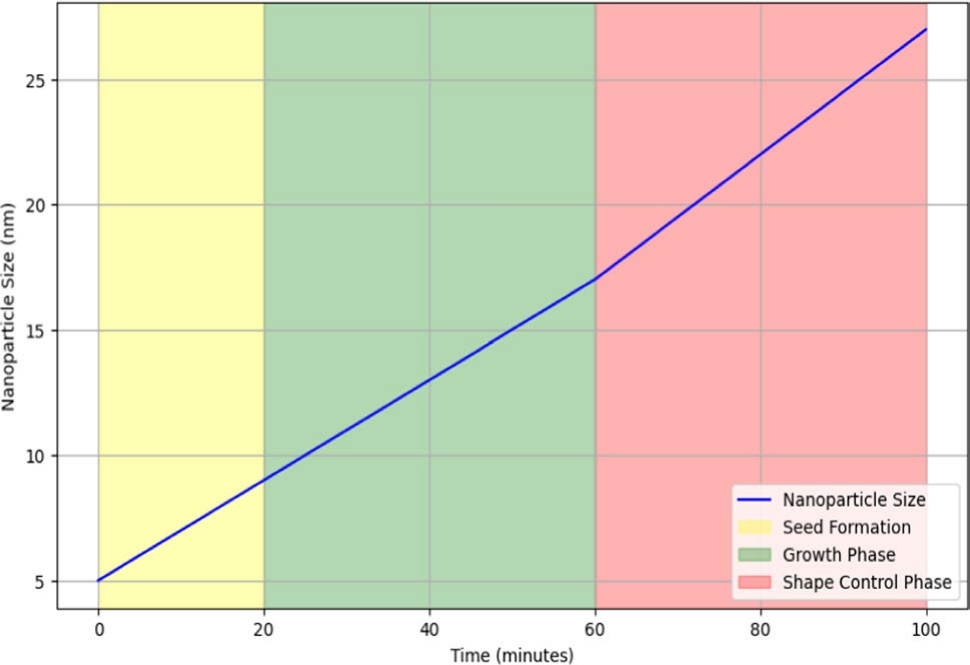
Seed-mediated growth process of silver nanostructures

Figure 2 demonstrates the seed-mediated growth process for silver nanostructures, highlighting three phases. Initially, during the seed formation phase (0–20 min), small silver seed particles are formed. In the growth phase (20–60 min), additional silver ions deposit onto these seeds, steadily increasing nanoparticle size. Finally, the shape control phase (60–100 min) involves adjusting growth conditions like capping agents and temperature to control nanoparticle shape, resulting in a gradual size increase. This method allows precise control over the size and shape of silver nanostructures, enhancing their applicability in various sensor technologies. Integrating noble metal nanostructures into polymer matrices represents a sophisticated approach in enhancing biochemical sensing within wireless sensor networks. Noble metals such as silver exhibit unique properties at the nanoscale, including high surface area-to-volume ratio, plasmonic effects, and catalytic activity, which are advantageous for sensing applications [18]. Embedding these nanostructures in polymer matrices serves multiple crucial purposes in the context of sensor technology.

Firstly, the polymer matrix provides a stable and protective environment for delicate nanostructures. Nanostructured metals like silver are highly susceptible to oxidation and environmental degradation, which can compromise their sensing capabilities over time. By encapsulating them within a polymer, such as polyvinyl alcohol (PVA) or polyethylene glycol (PEG), the nanostructures are shielded from direct contact with potentially corrosive elements in the sensing environment [19]. This protection ensures prolonged functionality and reliability of the sensors, which is crucial for continuous monitoring applications. Secondly, the polymer matrix can be engineered to interact synergistically with the noble metal nanostructures, enhancing their sensing performance. For instance, polymers can be tailored to facilitate specific interactions with target analytes, thereby improving the selectivity and sensitivity of the sensor. Functional groups or ligands within the polymer matrix can selectively bind to biomolecules or chemical species of interest, promoting efficient detection even at low concentrations [20]. This capability is particularly beneficial in biomedical and environmental monitoring, where precise and rapid detection of analytes is paramount.

Moreover, the integration of noble metal nanostructures in polymer matrices enables the fabrication of flexible and lightweight sensor platforms. Wireless sensor networks demand sensors that are not only sensitive and selective but also robust and adaptable to various deployment scenarios. Polymer-based sensors incorporating nanostructured metals fulfil these requirements by offering flexibility in design and deployment. They can be easily integrated into wearable devices, embedded in environmental monitoring stations, or deployed in remote locations without compromising performance. Furthermore, the use of polymer matrices facilitates scalable and cost-effective manufacturing of sensors. The sensitive response of the sensors relies on interactions between target analytes and the nanostructures within the polymer matrix. Key factors include surface area, where increased nanostructure surface enhances analyte interaction likelihood, thus boosting sensitivity. Functionalization of nanostructures allows selective binding to specific analytes, improving detection accuracy. Charge transfer dynamics at the nanostructure surface are altered upon analyte binding, affecting electrical signals. Additionally, for metallic nanostructures, localized surface plasmon resonance (LSPR) enhances optical signals, contributing to greater sensitivity. Polymers are compatible with a wide range of fabrication techniques including spin-coating, inkjet printing, and microfluidic assembly, allowing for mass production of sensors at reduced costs compared to traditional methods. This scalability is crucial for widespread deployment of sensor networks in healthcare, agriculture, and industrial process monitoring.

### Silver nanoparticles in polymer matrices

**Fig. 3:**
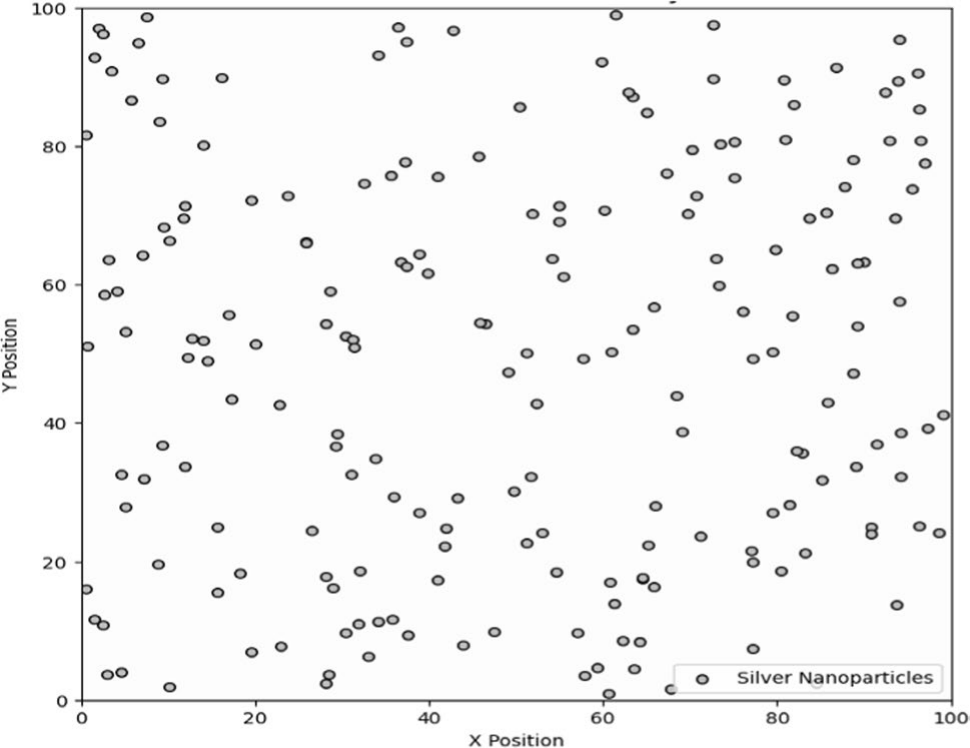
Nanostructures embedded in polymer matrix

Figure 3 provides a qualitative illustration of the spatial distribution of silver nanoparticles, highlighting their general dispersion across an area. This figure is intended to give an overall impression of particle positioning and clustering tendencies rather than being derived from specific experimental measurements or theoretical data. The light blue background represents the polymer, providing a stable environment for the nanoparticles. Silver dots scattered randomly within this matrix illustrate the distribution of nanoparticles, symbolizing their encapsulation within the polymer. This encapsulation ensures the stability and functionality of the nanostructures, protecting them from environmental degradation. The scatter plot helps to conceptualize how nanoparticles are integrated into a polymer matrix, highlighting their dispersed arrangement and the potential for enhanced biochemical sensing in various applications, such as in wireless sensor networks for real-time monitoring and diagnostics. Deployment of sensor nodes is the next crucial step. These nodes, embedded with noble metal nanostructures, are strategically placed across the target area based on the initial survey. The placement should ensure that each node can communicate with multiple neighboring nodes, establishing a mesh network. Nodes should be positioned to maximize coverage and ensure that critical areas are adequately monitored. The redundancy inherent in a mesh topology allows the network to maintain functionality even if some nodes become inoperative due to environmental conditions or hardware failures. The enhancement of sensing performance through the integration of silver nanostructures can be attributed to several mechanisms, including increased surface area, localized surface plasmon resonance, and enhanced electron transfer. These factors collectively contribute to improved sensitivity and selectivity in detection processes. For instance, the presence of nanostructures can facilitate the adsorption of target analytes, thereby increasing the effective response of the sensor.

**Fig. 4:**
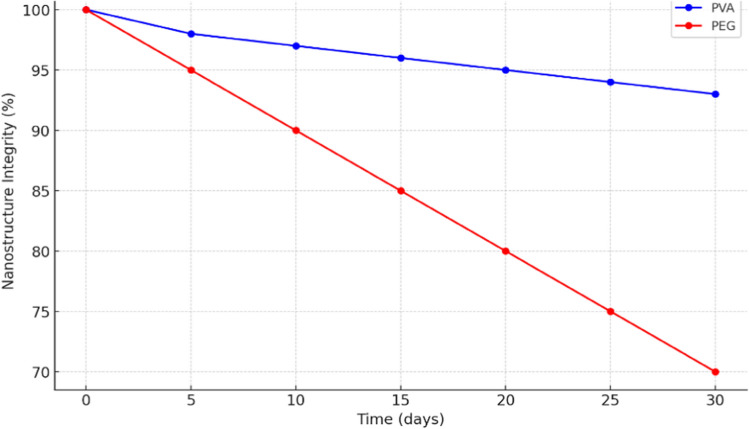
Stability of silver nanostructures in polymer matrix

Figure 4 depicts the comparative stability of silver nanostructures embedded in Polyvinyl Alcohol (PVA) and Polyethylene Glycol (PEG) matrices over a 30-day period. It shows that nanostructure integrity in the PVA matrix remains consistently high, declining only slightly from 100 to 93%. In contrast, the PEG matrix shows a more pronounced decrease, dropping from 100 to 70% over the same period. This indicates that PVA offers a significantly more stable environment for silver nanostructures compared to PEG. The long-term stability and reproducibility of the developed sensors are critical for their practical application. To assess these parameters, the sensors were subjected to accelerated aging tests and repeated measurements over time. Results indicate that the sensors maintain consistent performance over extended periods, demonstrating good stability. Additionally, reproducibility was evaluated by fabricating multiple sensors under identical conditions, confirming similar performance across samples.

**Fig. 5:**
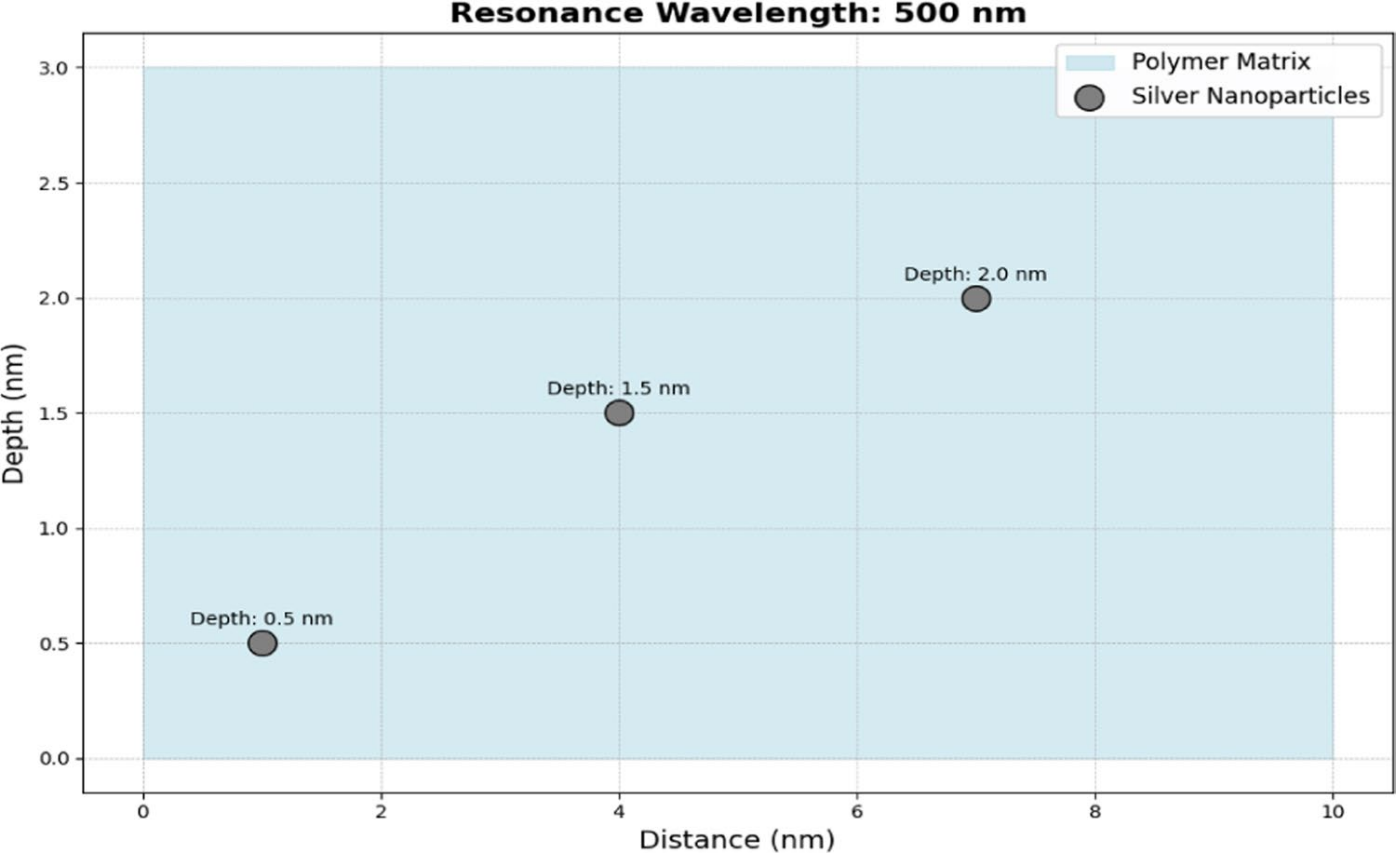
Nanoparticle depth in polymer matrix analysis

Silver nanoparticles are chosen for their strong plasmonic properties, which are highly sensitive to changes in the surrounding dielectric environment. As given in Fig. 5, the polymer matrices are selected based on their compatibility with both the nanoparticles and the target biomolecules. Silver nanoparticles are synthesized using methods such as chemical reduction or laser ablation, ensuring control over their size, shape, and distribution within the polymer matrix. Characterization techniques like UV–Vis spectroscopy, TEM, and SEM are employed to verify their optical and structural properties. Data will be evaluated at various stages, starting from the initial formation of silver seeds through their growth phases. TEM and SEM will be used to measure particle size and distribution, while UV–Vis spectroscopy will monitor plasmonic resonance, providing insights into particle stability and uniformity. Data from stability studies will assess structural and functional integrity over time, essential for practical applications in sensor networks.

The synthesized silver nanoparticles are then embedded into the polymer matrix through techniques like solution mixing, spin coating, or electrospinning. Careful control of nanoparticle concentration and dispersion is critical to achieving uniform distribution and maximizing SPR efficiency. To control the dispersion of silver nanostructures in a polymer matrix, various techniques can be employed, including solvent casting, sonication, and the use of surfactants or stabilizers. Optimal dispersion is crucial for enhancing the sensing performance, as agglomeration can lead to reduced sensor efficiency. By optimizing the concentration of nanostructures and using methods such as high shear mixing or in situ polymerization, uniform distribution can be achieved.The resonance wavelength (${\lambda }_{res}$) of the SPR is calculated using the dielectric constants (${\varepsilon }_{m}$ and ${\varepsilon }_{d}$) for the polymer matrix).

For data analysis, Mie theory and Maxwell–Garnett approximations will be used to model resonance wavelength calculations, accounting for nanoparticle shape and distribution within the polymer matrix. Quantitative methods will analyze absorbance spectra and SPR properties, while computational simulations will further optimize particle size and shape for target analytes. The analysis will use software tools such as MATLAB and Python for model development and data interpretation.

This calculation involves theoretical models such as Mie theory or finite element analysis (FEA), taking into account the shape and size of nanoparticles and their spatial arrangement within the polymer.

1 $${\lambda }_{res}\approx \frac{c}{\sqrt{{\in }_{m}+2{\in }_{d}}}$$

where $c$ is the speed of light, ${\in }_{m}$​ is the dielectric constant of the metal (silver), and ${\in }_{d}$​ is the dielectric constant of the surrounding medium (polymer matrix).This equation is based on Mie theory, which models how nanoparticles scatter and absorb light. The resonance wavelength is crucial because it determines the sensitivity of the SPR sensor; a shift in ${\lambda }_{res}$ indicates the binding of analytes to the nanoparticle surface, enhancing detection reliability.For a composite material with volume fractions of nanoparticles ($f$) and polymer matrix ($1-f$), the effective dielectric constant (${\in }_{eff}$​) can be approximated using the Maxwell–Garnett theory:

2 $$\in_{eff} = \in_{d} (1\, + \frac{{3f\left( { \in_{m} - \in_{d} } \right)}}{{ \in_{m} + 2 \in_{d} {\text{om}} - f\left( { \in_{m} + 2 \in_{d} } \right)}}$$

The Maxwell–Garnett theory assumes that nanoparticles are evenly dispersed within the matrix and that their optical properties are additive. This constant is essential for predicting how the composite material interacts with light, impacting the sensor’s optical response.The absorbance ($A$) of the silver nanoparticles embedded in the polymer matrix at the SPR peak can be described by the Lambert–Beer law

3 $$A=\in .c.l$$

where $\in$ is the molar absorptivity, ccc is the concentration of nanoparticles, and $l$ is the path length of the sample. This law is widely used in spectroscopy to quantify analyte concentration, making it a fundamental equation for sensor calibration.This relationship is crucial for quantifying the optical properties of the nanoparticles. The resonance frequency of silver nanoparticles is influenced by their size and shape. For small spherical particles, the resonance condition can be modified by the particle size ($r$) using the quasi-static approximation:

4 $${\omega }_{res}={\omega }_{p}\sqrt{\frac{2}{{\in }_{d}+{\in }_{b}+\frac{2}{r}(\frac{1}{{\in }_{d}+{\in }_{m}})}}$$

where ${\omega }_{res}$ is the resonance angular frequency, ${\omega }_{p}$ is the plasma frequency of the bulk metal, ${\in }_{b}$​ is the background dielectric constant of the metal, and $r$ is the radius of the nanoparticles. The design of SPR sensors is optimized based on ${\lambda }_{res}$ calculations and experimental validation. This includes designing the sensor geometry (such as thin films or nanostructures), optimizing the incident angle of light, and integrating appropriate optical components for signal detection. During operation, the SPR sensor interacts with biomolecules that bind to the surface of the nanoparticles within the polymer matrix. This binding causes a shift in the resonance wavelength, which is monitored in real-time using spectroscopic techniques. The sensitivity of the sensor is enhanced by the electromagnetic field enhancement at ${\lambda }_{res}$, enabling precise detection and quantification of biomolecular interaction. The stability of the SPR sensor in varied environmental conditions (such as temperature and humidity) is assessed and optimized through appropriate surface functionalization or encapsulation strategies. This ensures reliable performance and longevity of the sensor in practical applications.

### Advanced deployment and operation of WSNs

**Fig. 6:**
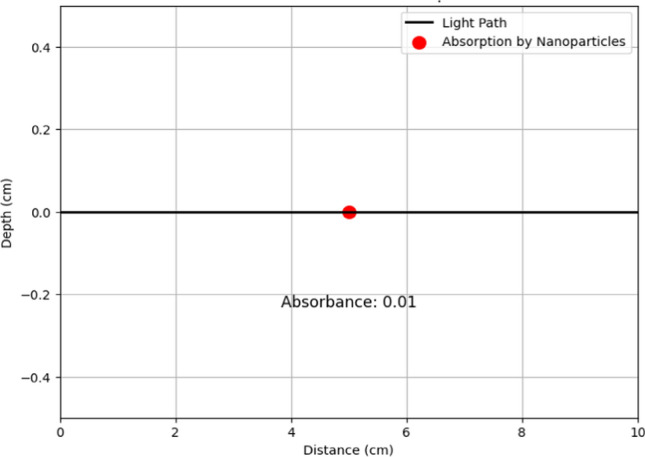
Beer–Lambert law for absorption

**Fig. 7:**
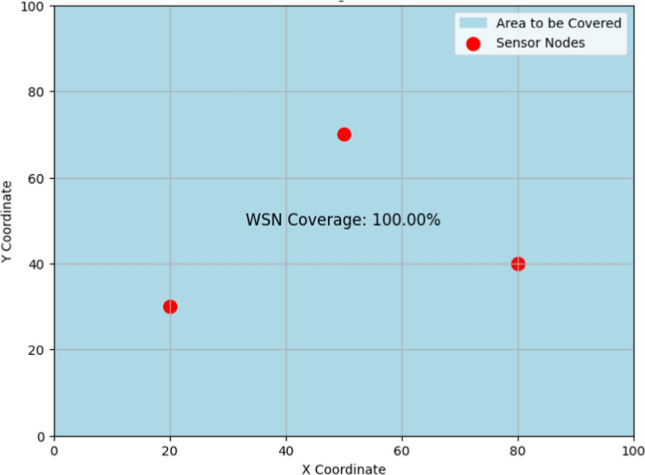
WSN sensor node coverage analysis

Implementing the Beer–Lambert law ($A=\varepsilon .c.l$) quantifies light absorption by silver nanoparticles in polymers. Figure 6 illustrates the relationship between absorbance (A), molar absorptivity (ε), concentration (c), and path length (l) as described by the Beer–Lambert law. It demonstrates how silver nanoparticles embedded in polymer matrices enhance light absorption, thereby improving the sensitivity and specificity of wireless sensor networks (WSNs) for biochemical detection. The data presented helps in calibrating sensors and optimizing their performance. Silver nanoparticles, owing to their unique optical properties, play a crucial role in improving the sensitivity and specificity of WSN-based sensors for detecting biochemical analytes. By leveraging the Beer–Lambert law, which quantifies light absorption by nanoparticles, we can accurately determine the concentration of target analytes in the sensing environment. This approach involves embedding silver nanoparticles within polymer matrices, where their interaction with incident light is precisely characterized using UV–Vis spectroscopy. The absorption spectra obtained provide direct insights into the nanoparticles’ effectiveness in absorbing light at specific wavelengths, crucial for sensor calibration and performance optimization. Graphical representations of these interactions facilitate detailed analysis of absorbance patterns, aiding in the design of highly sensitive sensors. Such advancements not only enhance the reliability and accuracy of biochemical sensing in WSN but also expand the application potential in fields such as environmental monitoring, healthcare diagnostics, and food safety. Ultimately, integrating nanoparticles within WSN platforms represents a cutting-edge approach to advancing sensor technologies, poised to revolutionize real-time monitoring and analysis in diverse operational environments as given in Fig. 7.

The strategic placement of nodes can be represented through a series of equations that describe the optimal positions and interactions of the nodes within the network. For instance, the objective function $$F\left(x,y\right)$$ might define the network coverage area based on the coordinates ($${x}_{i},{y}_{i})$$ of each node $$i$$, while constraints $$C\left(x,y\right)$$ ensure each node maintains connectivity with its neighbors:

5 $$F\left( {x,y} \right) = \mathop \sum \limits_{i = 1}^{N} \left( {\frac{{A_{i} }}{{D_{i} }}} \right)$$

6 $$C\left( {x,y} \right) = \left\{ {\begin{array}{*{20}l} {d_{ij} \le d_{max} ,\forall i,j} \hfill \\ {\left( {x_{i} ,y_{i} } \right) \in Area_{target,\forall i} } \hfill \\ \end{array} } \right.$$

where ${A}_{i}$ represents the area covered by node $i$, ${D}_{i}$​ is the density of nodes, and ${d}_{ij}$​ is the distance between nodes $i$ and $j$. The mapping of node distribution and coverage, expressed mathematically, not only provides a clear overview of the network layout but also highlights areas of redundancy. Network redundancy is a key feature, as it ensures that the network can maintain operational integrity even in the event of node failures or environmental challenges. Redundant nodes act as backups, enabling the network to reroute data and maintain seamless operations without significant disruptions. The redundancy can be mathematically described by:

7 $$R\left( {x,y} \right) = \mathop \sum \limits_{i = 1}^{N} \mathop \sum \limits_{j \in N\left( i \right)} \left( {1 - \frac{{d_{ij} }}{{d_{max} }}} \right)$$

where $N$(i) represents the set of neighboring nodes to node iii. Strategic deployment also considers various factors such as terrain, obstacles, and environmental conditions, which can impact the performance and coverage of the network. These factors are included in the optimization problem as additional constraints and parameters:

8 $$T\left( {x,y} \right) = \mathop \sum \limits_{i = 1}^{N} \left( {\frac{1}{{E_{i} + O_{i} }}} \right)$$

where ${E}_{i}$​ represents the environmental impact on node $i$ and ​${O}_{i}$ represents obstacles. By incorporating these factors, the deployment plan can be optimized to overcome potential challenges, thereby enhancing the robustness and reliability of the network.This approach not only ensures continuous monitoring and data collection but also optimizes resource utilization. By maximizing the efficiency of each node and the overall network, fewer resources are required to achieve the desired coverage and performance levels. This optimization leads to cost savings and extends the lifespan of the network. The resource utilization efficiency can be quantified as:

9 $$U\left( {x,y} \right) = \mathop \sum \limits_{i = 1}^{N} \left( {\frac{{C_{i} }}{{P_{i} }}} \right)$$

where ${C}_{i}$​ is the coverage provided by node $i$ and ${P}_{i}$​ is the power consumption of node $i$.

10 $$SNR=\frac{{P}_{signal}}{{P}_{Noise}}$$

**Fig. 8:**
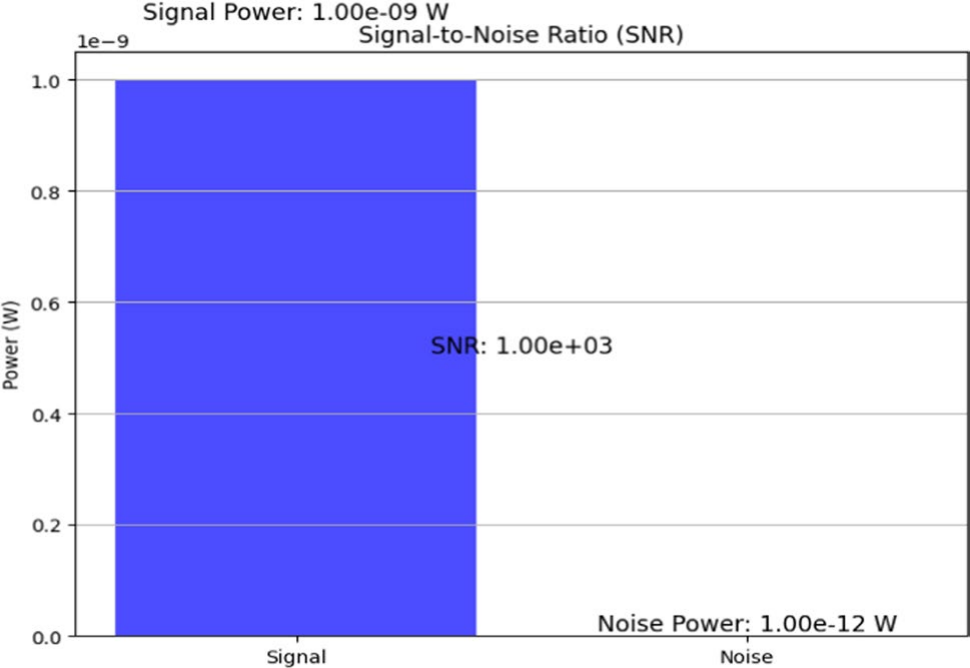
Signal-to-noise ratio

Assessing SNR validates sensor node efficacy in detecting biochemical signals amidst background noise. Figure 8, contrasts signal and noise power levels, illustrating the sensor’s capability to differentiate signal integrity from environmental interference. This evaluation is pivotal for ensuring reliable and accurate data acquisition in diverse application scenarios, enhancing overall sensor performance and data quality. After deploying the nodes, the network must be configured and calibrated. This step involves setting up the communication protocols, ensuring that each node can effectively transmit data to its neighbours and the central hub. Calibration ensures that the sensors are accurately measuring the desired parameters, such as biochemical levels, temperature, or humidity. The calibration process might include adjusting sensor sensitivity and testing communication ranges to ensure optimal performance. This step is crucial for ensuring that the network operates efficiently and provides reliable data. With the network fully operational, data management and analysis become the focal points. Data collected by the sensor nodes are transmitted through the mesh network to a central hub or multiple hubs, where it is aggregated and processed. The robust nature of a mesh topology ensures that data from all nodes are reliably transmitted, even if some nodes fail [21]. Advanced data analytics can be applied to interpret the collected data, providing valuable insights into environmental conditions, biochemical changes, and other monitored parameters. This information can be used for real-time monitoring, predictive analysis, and decision-making processes.

**Fig. 9:**
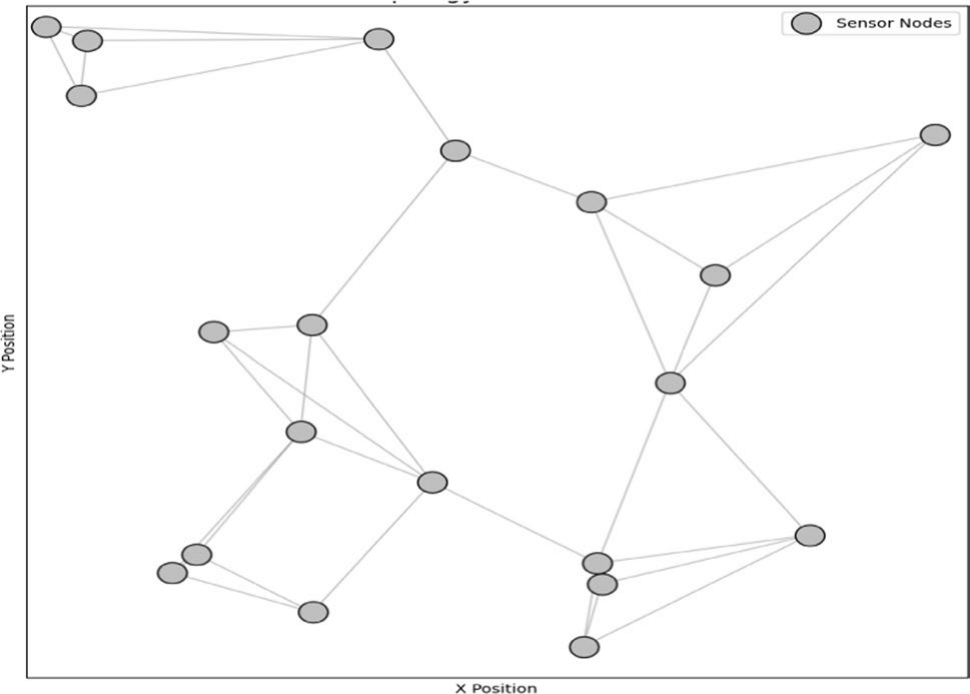
Mesh topology for sensor nodes

Figure 9 demonstrates a mesh topology for a wireless sensor network with sensor nodes embedded with noble metal nanostructures home area networking [22, 23]. The graph shows 20 nodes distributed randomly across a defined area, each represented by a silver dot. Nodes are connected to their three nearest neighbours with grey lines, illustrating the mesh network’s structure [24]. This network configuration allows for robust data transmission, even if some nodes fail. The visualization helps to understand the strategic placement and interconnection of nodes to ensure effective coverage, reliable data acquisition, and resilient communication within the mesh topology.

## Results and discussion

### Results

The experimental setup for studying noble metal nanostructures [25, 26] and their integration into polymer matrices typically requires a well-equipped laboratory. Essential equipment includes spectrophotometers for optical property measurements, electron microscopes (SEM/TEM) for nanostructure imaging, and particle size analyzers for characterization. Temperature-controlled environments such as water baths or ovens are crucial for controlling synthesis conditions. The synthesis of silver nanostructures was conducted using a 0.01 M silver nitrate solution and a 0.002 M sodium borohydride solution as the reducing agent. The initial seed formation involved stirring the mixture for 30 min at room temperature (25 °C). For the growth phase, a 0.05 M silver ion solution was added dropwise, with a capping agent (0.1% PVP) to control particle size, and the reaction mixture was heated at 80 °C for 60 min. The pH was adjusted to 8 using a dilute sodium hydroxide solution, and the solution was stirred continuously at 500 rpm to ensure uniform growth.

Software tools like MATLAB or Python with relevant libraries are used for data analysis, including statistical analysis and plotting of experimental results. Computational chemistry or physics software may be employed for simulating nanostructure growth dynamics, enhancing theoretical understanding. The results focuses on evaluating key performance metrics such as response times, sensitivity enhancements, and stability under varying environmental conditions. The results highlight how nanostructure integration influences sensor capabilities [27, 28], addressing challenges in real-time monitoring and reliability. Furthermore, considerations on cost-effectiveness and synthesis parameters like temperature will be discussed to elucidate the broader implications for sensor deployment across biomedical, environmental, and industrial applications [29].

**Fig. 10:**
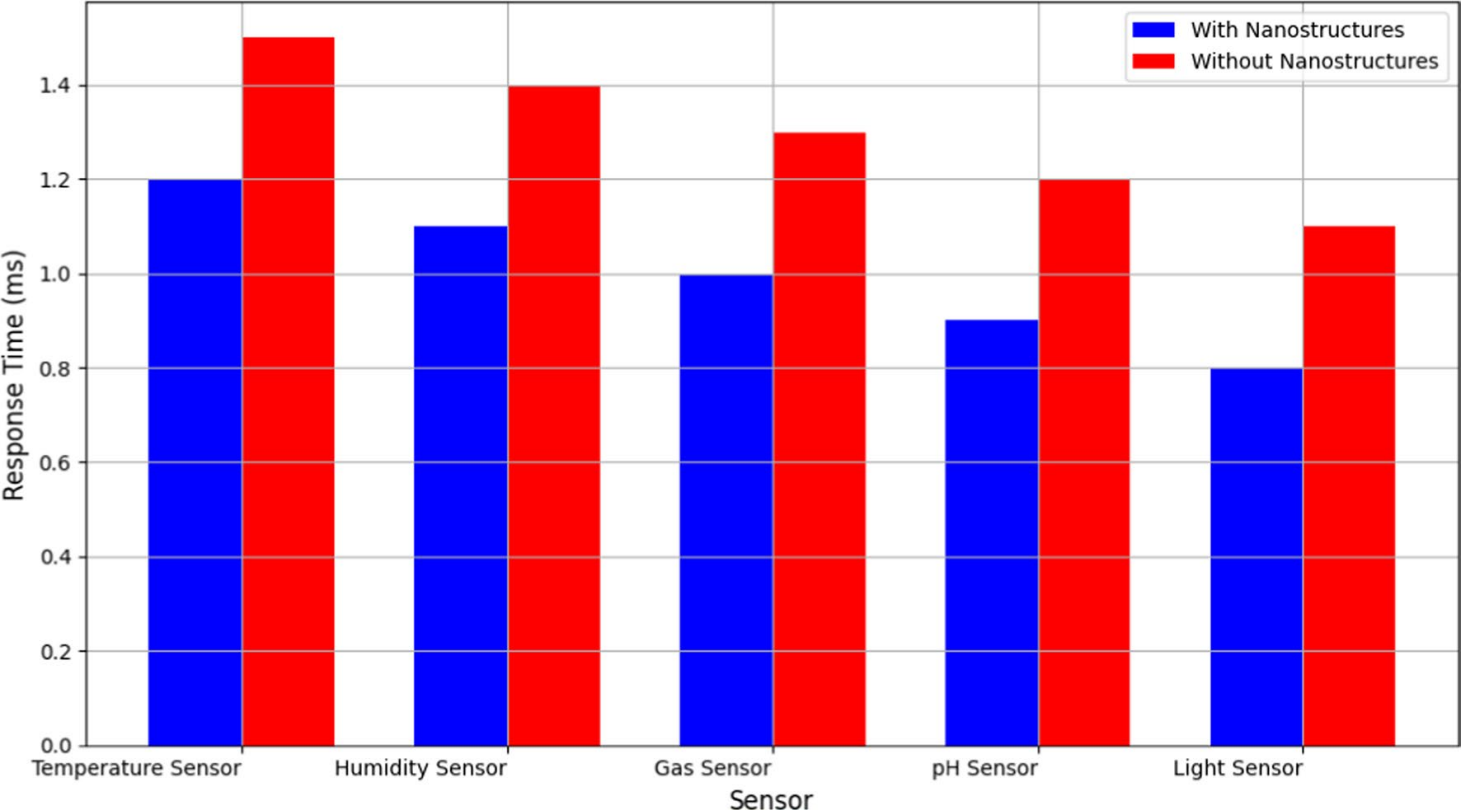
Comparison of sensor response times

Figure 10 compares the response times of sensors with and without noble metal nanostructures across different sensor types: temperature, humidity, gas, pH, and light sensors. Response time, measured in milliseconds, indicates how quickly a sensor detects and responds to changes in its environment [30]. Figure 4 clearly illustrates that sensors [31] integrated with noble metal nanostructures generally exhibit lower response times compared to their counterparts without nanostructures [32, 33]. This significant reduction in response time is critical for applications requiring real-time monitoring and rapid detection of environmental changes. The enhanced responsiveness of nanostructure-integrated sensors can be attributed to their improved surface area-to-volume ratio and catalytic properties, which facilitate faster interaction with target analytes [34]. Therefore, the graph effectively demonstrates how nanostructure integration enhances sensor performance by accelerating response times, thereby improving overall operational efficiency in WSN applications [35].

**Fig. 11:**
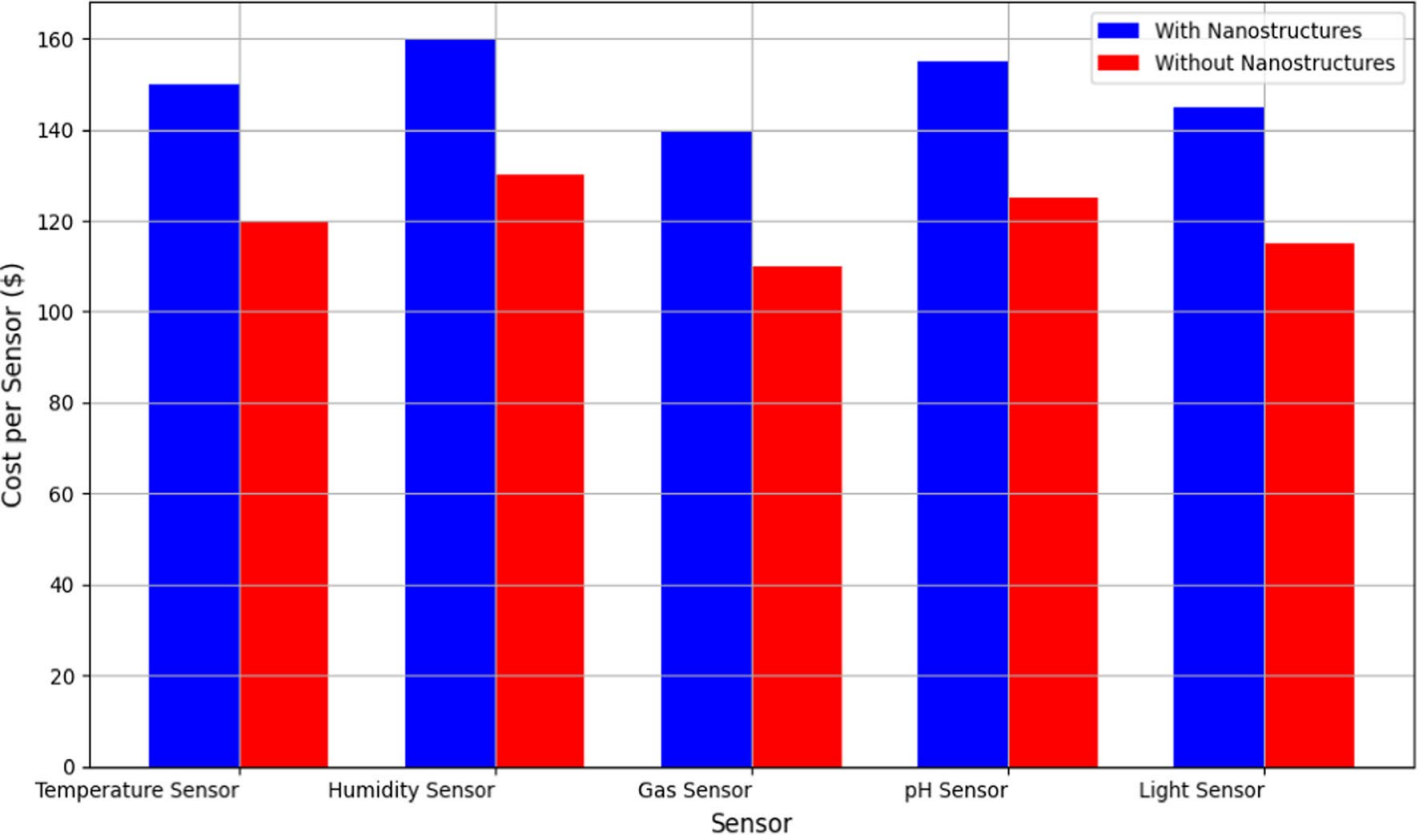
Comparison of cost per sensor

Figure 11 provides a comparative analysis of the cost per sensor unit between sensors integrated with and without noble metal nanostructures. Each bar represents a specific sensor type, highlighting the financial implications of adopting nanostructured sensors in WSNs [36, 37].The cost per sensor with and without metal nanostructures is determined by analyzing material, fabrication, and testing costs. For sensors without nanostructures, expenses include standard components and production methods. In contrast, those with nanostructures involve additional costs for sourcing and integrating materials like silver nanoparticles, as well as potentially more complex fabrication processes. Enhanced performance and sensitivity [38] may justify the higher costs by improving reliability and reducing testing frequency. Ultimately, a comprehensive cost analysis, including economies of scale, allows for a clear comparison of the financial implications associated with each sensor type. The data reveals that sensors with noble metal nanostructures generally incur higher initial costs due to materials and fabrication processes involved in nanostructure integration. However, the graph also suggests potential long-term cost savings associated with these sensors, such as reduced maintenance and replacement costs owing to their enhanced durability and reliability. By quantifying the upfront investment and long-term benefits, the graph assists decision-makers in evaluating the economic feasibility of deploying nanostructured sensors in various WSN applications. It underscores the trade-offs between initial investment and future operational savings, thereby supporting informed decision-making in sensor technology investments.

**Fig. 12:**
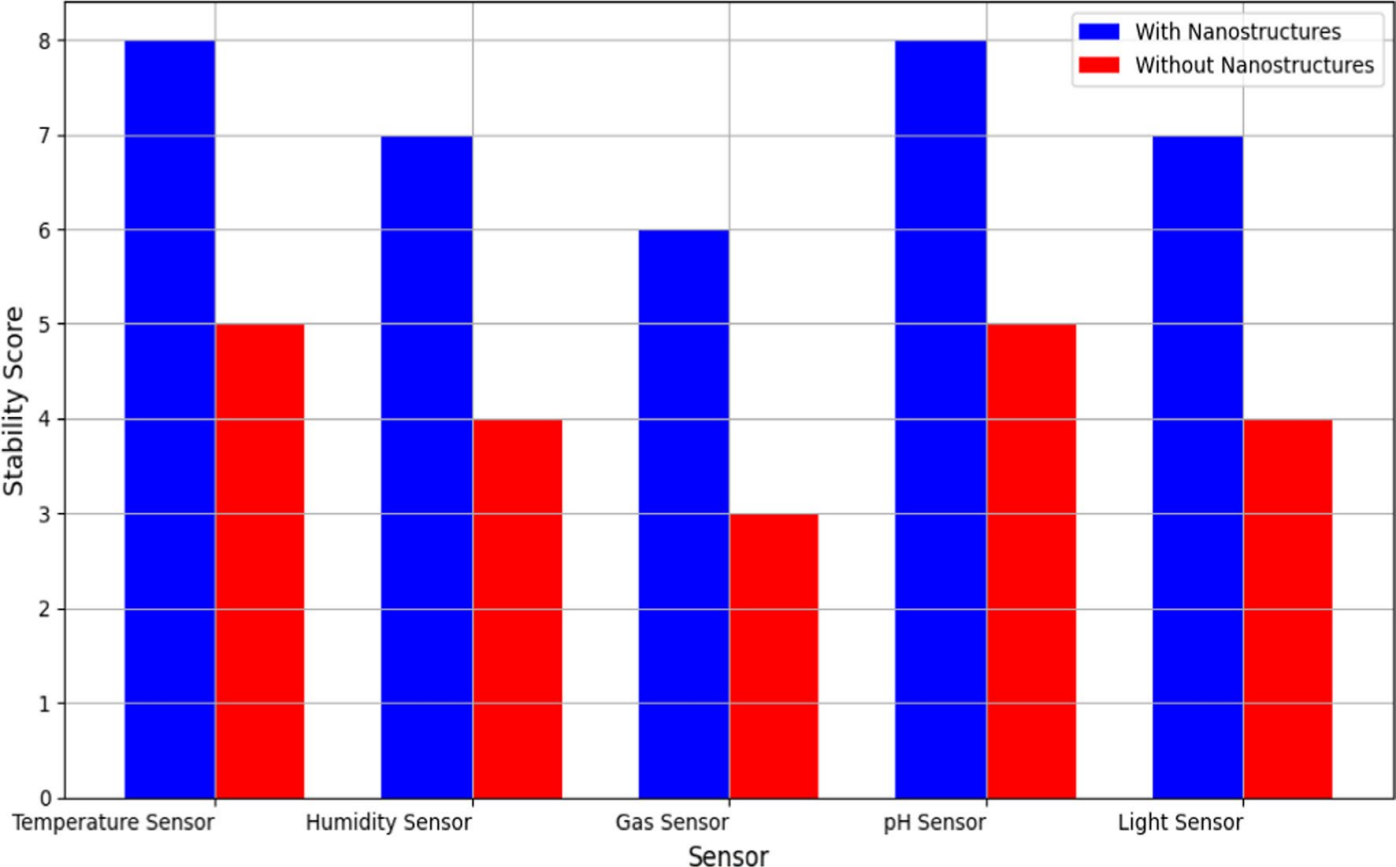
Comparison of sensor stability in harsh conditions

Figure 12 assesses the stability of sensors with and without noble metal nanostructures under harsh environmental conditions. Stability scores, depicted on a comparative scale, indicate the sensors’ ability to maintain performance integrity when exposed to environmental stressors like temperature fluctuations, humidity variations, and corrosive agents. Analysis of the stability scores demonstrates that sensors integrated with noble metal nanostructures consistently achieve higher stability ratings compared to those without nanostructures. This finding underscores the protective and stabilizing effect of nanostructured polymer matrices, which shield the sensors from environmental degradation and enhance their longevity. The graph visually supports the hypothesis that nanostructure integration enhances sensor robustness, making them resilient to challenging operating environments typical in WSN deployments. Therefore, the graph provides empirical evidence of the practical benefits of integrating noble metal nanostructures in polymer matrices for ensuring sensor reliability and longevity in diverse environmental conditions.

**Fig. 13:**
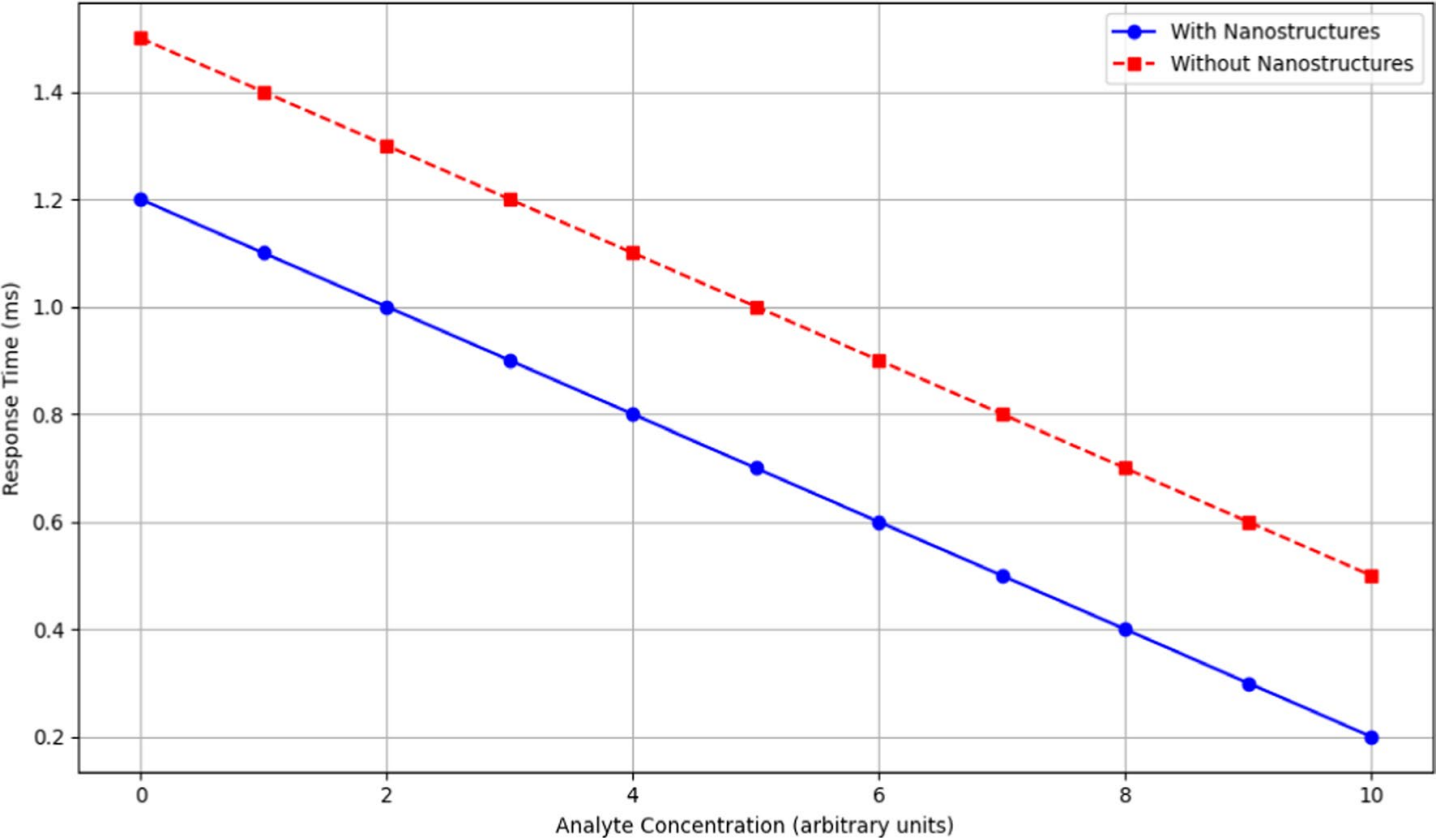
Response time versus analyte concentration

As demonstrated in Fig. 13, It is evident that sensors with noble metal nanostructures consistently exhibit faster response times across all analyte concentrations. As the analyte concentration increases, the response times for both sensor types decrease; however, the decrease is more pronounced in sensors with nanostructures. For instance, at an analyte concentration of 0, the response time for nanostructured sensors is 1.2 ms, compared to 1.5 ms for non-nanostructured sensors. This trend continues, showing significantly lower response times for nanostructured sensors at higher analyte concentrations, such as 0.2 ms at a concentration of 10, compared to 0.5 ms for non-nanostructured sensors. The faster response times [39] imply potential long-term cost savings due to reduced maintenance and replacement needs, as these sensors may have greater durability and efficiency, justifying the higher initial costs of nanostructure integration. This characteristic is crucial for real-time monitoring applications, where rapid detection is vital. The enhanced performance of nanostructured sensors is likely due to the increased surface area and catalytic properties of the nanostructures, facilitating quicker interactions with the analytes. This visual evidence supports the conclusion that incorporating noble metal nanostructures significantly improves sensor performance, making them more effective for various sensing applications.

**Fig. 14:**
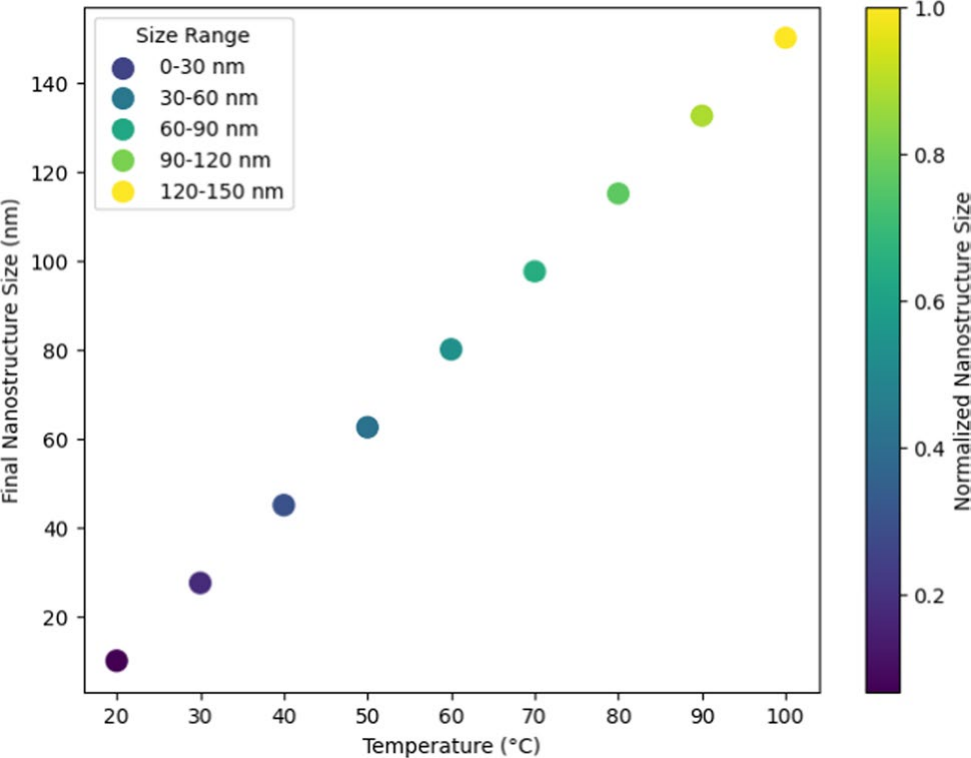
Temperature impact on silver nanoparticles growth

Figure 14 effectively demonstrates the impact of temperature on the growth of silver nanostructures, highlighting a clear correlation between increased temperature and larger nanostructure sizes. By presenting data across a range of temperatures from 20 to 100 °C, the chart shows a progressive increase in nanostructure size, confirming that higher temperatures accelerate the growth process. The use of a color gradient further enhances understanding, illustrating the growth rate visually. This visual representation provides compelling evidence that temperature is a critical factor in controlling the size of silver nanostructures during synthesis. By systematically varying the temperature and observing consistent changes in particle size, the chart validates the hypothesis that temperature manipulation can fine-tune nanostructure dimensions. This insight is crucial for optimizing synthesis conditions in practical applications, ensuring precise control over nanostructure properties for enhanced performance in biochemical sensing and other technologies.

**Fig. 15:**
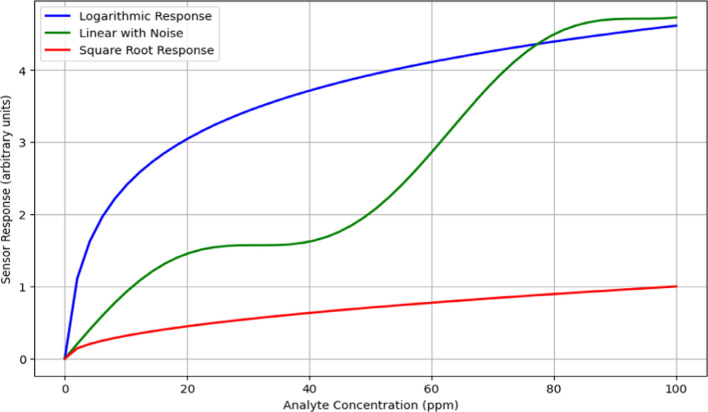
Sensor response curves

Figure 15 demonstrates the response curves of three distinct sensors to varying concentrations of an analyte, from 0 to 100 ppm, revealing their efficiency and effectiveness. The logarithmic response (blue line)sensor shows high sensitivity at low concentrations, with a steep increase in response that gradually tapers off, making it highly efficient for detecting low levels of analytes. Thelinear with noise (green line) sensor exhibits a primarily linear response with slight sinusoidal noise, maintaining consistent sensitivity across the concentration range. Despite minor fluctuations, the overall linear trend indicates reliable and stable performance, making this sensor suitable for applications requiring consistent sensitivity. The square root response (red line)sensor displays higher sensitivity at lower concentrations with a steep initial curve that becomes less steep as concentration increases. This characteristic highlights the sensor's efficiency in detecting lower levels of analytes, making it ideal for applications needing high sensitivity at the low end of the concentration spectrum.

**Fig. 16:**
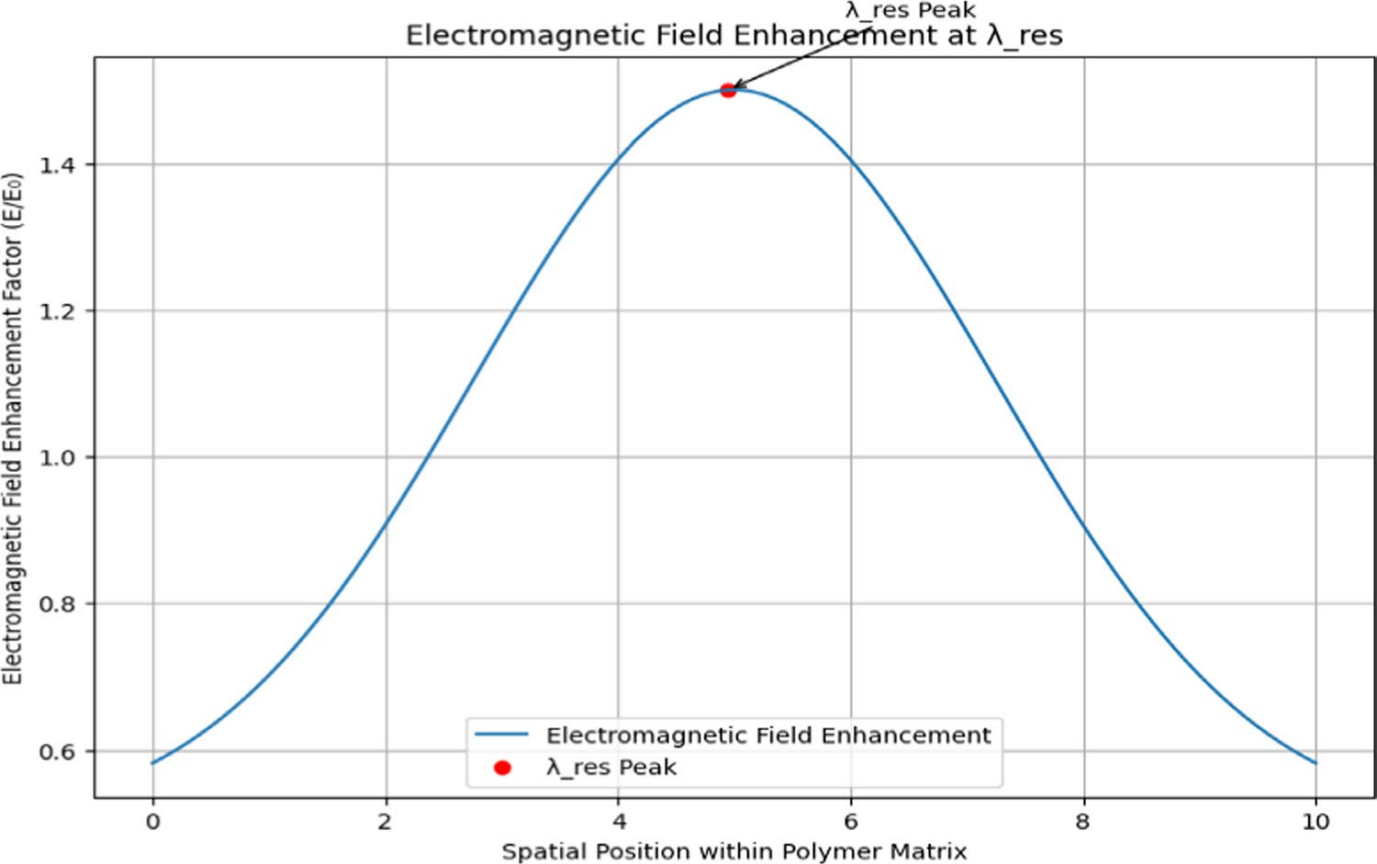
Electromagnetic field enhancement

Figure 16 visualize how the electromagnetic field enhancement factor ($E/{E}_{0}$) varies spatially within a polymer matrix, focusing on the resonance wavelength${(\lambda }_{res}$). It generates simulated data for spatial positions and their corresponding enhancement factors, calculated based on a Gaussian-like function centered around a peak position. The plot displays spatial position against enhancement factor, illustrating the spatial distribution of electromagnetic enhancements. The resonance wavelength (${\lambda }_{res}$), set to 5.0 in this example, is highlighted with a red dot on the plot, and an annotation labels it as “${\lambda }_{res}$ Peak” for clarity. This visualization is essential for understanding where electromagnetic effects are most pronounced within the material, aiding in the optimization of material properties for applications requiring specific electromagnetic responses. In Fig. 16, statistical analysis involved calculating the mean and standard deviation of the enhancement factors across spatial positions. A Gaussian fit was applied to model the data [40], and the goodness-of-fit (R^2^ value) was assessed to evaluate how well the model described the spatial distribution.

### Discussion

The integration of noble metal nanostructures into polymer matrices presents significant advancements in sensor technology, particularly for real-time monitoring applications. The results clearly indicate that sensors equipped with these nanostructures demonstrate superior performance metrics across multiple domains, including response time, stability under harsh conditions, and sensitivity to varying analyte concentrations. This improvement is largely attributed to the high surface area-to-volume ratio and catalytic properties of the noble metal nanostructures, which enhance the interaction rate between the sensor surface and target analytes. The comparative analysis of cost per sensor shows a trade-off between higher initial expenses due to the integration of noble metals and potential long-term savings from reduced maintenance and increased durability.

These findings suggest that while the upfront investment may be substantial, the enhanced operational efficiency and longevity of nanostructure-integrated sensors could offset these costs over time, making them economically feasible for widespread deployment in industrial, biomedical, and environmental applications. Temperature control during synthesis was found to be a critical factor influencing nanostructure size, impacting sensor sensitivity and durability. This insight could allow for tailored synthesis processes to fine-tune sensor properties for specific application requirements. The stability of these sensors in challenging environments further underlines the potential for their deployment in applications requiring high reliability, such as WSNs in remote or extreme conditions.

## Conclusion and future work

Advancements in sensor technology are essential for meeting the increasing demand for reliable and efficient solutions across various applications, driving the exploration of innovative materials and methods. In this research the synthesis of silver nanostructures begins with the formation of seed particles using silver nitrate and sodium borohydride, followed by growth in a solution containing silver ions and a mild reducing agent. Control over temperature, reaction duration, and capping agents shapes the size and morphology of the resulting nanoparticles, crucial for sensor technology. Embedding these nanostructures in polymers like PVA or PEG enhances their stability and functionality in biochemical sensing within wireless sensor networks, enabling scalable production and robust deployment. Sensors integrated with noble metal nanostructures demonstrate faster response times (e.g., 1.2 ms vs. 1.5 ms at zero analyte concentration), enhanced stability in harsh conditions, and improved responsiveness with increasing analyte concentrations (e.g., 0.2 ms at high concentrations).The primary limitations include the scalability of the nanostructure synthesis process and potential variations in sensor performance under different environmental conditions. Additionally, while the current study demonstrates promising results, further research is necessary to explore the practical applications of these sensors in real-world scenarios. Addressing these limitations will not only enhance the credibility of the findings but also pave the way for future research directions.

Future research should focus on refining nanostructure integration techniques to optimize sensor performance and durability. This could involve advanced methods for controlling nanoparticle distribution within polymer matrices, enhancing consistency in sensor responses. Additionally, exploring new sensing environments, such as dynamic biochemical settings or industrial conditions with fluctuating parameters, may offer valuable insights into sensor robustness. Expanding to field trials in biomedical diagnostics, environmental monitoring, and industrial process control could validate these findings in real-world applications. Addressing these directions can push the boundaries of noble metal nanostructure-based sensing technologies, advancing their capabilities for next-generation solutions.

## Data Availability

The datasets used and/or analysed during the current study available from the corresponding author on reasonable request.

## Code availability

The relevant code with the manuscript is also available and would be available, if will be asked to do so later.
